# Increased Carrier Peptide Stability through pH Adjustment Improves Insulin and PTH(1-34) Delivery In Vitro and In Vivo Rather than by Enforced Carrier Peptide-Cargo Complexation

**DOI:** 10.3390/pharmaceutics12100993

**Published:** 2020-10-20

**Authors:** Mie Kristensen, Ragna Guldsmed Diedrichsen, Valeria Vetri, Vito Foderà, Hanne Mørck Nielsen

**Affiliations:** 1 Department of Pharmacy, Faculty of Health and Medical Sciences, University of Copenhagen, Universitetsparken 2, DK-2100 Copenhagen, Denmark; ragna.diedrichsen@sund.ku.dk (R.G.D.); vito.fodera@sund.ku.dk (V.F.); 2 Center for Biopharmaceuticals and Biobarriers in Drug Delivery, University of Copenhagen, Universitetsparken 2, DK-2100 Copenhagen, Denmark; 3 Dipartimento di Fisica e Chimica, Università Degli Studi di Palermo, Viale delle Scienze ed. 18, IT-90128 Palermo, Italy; valeria.vetri@unipa.it

**Keywords:** intestinal peptide delivery, insulin, PTH(1-34), cell-penetrating peptide, penetratin, carrier peptide

## Abstract

Oral delivery of therapeutic peptides is hampered by their large molecular size and labile nature, thus limiting their permeation across the intestinal epithelium. Promising approaches to overcome the latter include co-administration with carrier peptides. In this study, the cell-penetrating peptide penetratin was employed to investigate effects of co-administration with insulin and the pharmacologically active part of parathyroid hormone (PTH(1-34)) at pH 5, 6.5, and 7.4 with respect to complexation, enzymatic stability, and transepithelial permeation of the therapeutic peptide in vitro and in vivo. Complex formation between insulin or PTH(1-34) and penetratin was pH-dependent. Micron-sized complexes dominated in the samples prepared at pH-values at which penetratin interacts electrostatically with the therapeutic peptide. The association efficiency was more pronounced between insulin and penetratin than between PTH(1-34) and penetratin. Despite the high degree of complexation, penetratin retained its membrane activity when applied to liposomal structures. The enzymatic stability of penetratin during incubation on polarized Caco-2 cell monolayers was pH-dependent with a prolonged half-live determined at pH 5 when compared to pH 6.5 and 7.4. Also, the penetratin-mediated transepithelial permeation of insulin and PTH(1-34) was increased in vitro and in vivo upon lowering the sample pH from 7.4 or 6.5 to 5. Thus, the formation of penetratin-cargo complexes with several molecular entities is not prerequisite for penetratin-mediated transepithelial permeation a therapeutic peptide. Rather, a sample pH, which improves the penetratin stability, appears to optimize the penetratin-mediated transepithelial permeation of insulin and PTH(1-34).

## 1. Introduction

Therapeutic peptides are today widely used due to their high specificity giving more predictive responses and fewer side effects than conventional synthetic small molecule drugs. Because of the physico-chemical characteristics of peptide-based therapeutics, such as their proteolytic instability, hydrophilic nature, and a large molecular size, the mode of administration is mainly via injections. However, non-injectable delivery would in many cases be the preferred route when taking patient compliance into consideration.

To increase the transepithelial permeation of therapeutic peptide drugs, cell-penetrating peptides (CPPs) has shown promising potential as carriers [1,2]. A widely used methodology is to co-administer the therapeutic peptide with the CPP as a physical mixture. Co-administration eases sample preparation and enables optimization of the drug-to-CPP molar ratio when compared to covalent conjugation of the drug to the CPP. Also, the co-administration approach possibly lowers the risk of negatively affecting the biological activity of the drug and the cell-penetrating efficiency of the CPP when compared to covalent conjugation [3]. The co-administration approach may, however, result in the formation of a pool of poorly defined complexes differing in size and morphology [4,5]. On the other hand, complex formation may have beneficial effects such as serving as protection against enzymatic degradation [6] or facilitate high local concentrations of CPP and the cargo drug at the site of absorption in the intestine.

So far, studies investigating the effects of complexation between carrier peptides and therapeutic peptides in order to obtain sufficient transepithelial permeation of the latter are limited. Previous studies have concluded that electrostatic interactions between a CPP and a co-administered therapeutic peptide or protein are important for CPP-mediated transepithelial delivery of the cargo [7,8,9]. In contrast, another study demonstrated that the CPP penetratin [10] mediated transepithelial insulin permeation mainly at pH-values which did not favor complexation between insulin and penetratin [11]. With the present study, we will provide further knowledge to explain the effects of intermolecular interactions and sample pH when exploiting CPPs as carriers for transepithelial permeation of co-administered therapeutic peptides.

In the present study, insulin and the pharmacologically active part of parathyroid hormone (PTH(1-34)) [12] were included as relevant therapeutic peptides, differing in isoelectric points and molecular weights. The cationic 16-mer penetratin sequence was employed as carrier. Penetratin has earlier shown potential for improving the transepithelial delivery of co-administered cargo peptides [13]. pH 5, 6.5, and 7.4 were included as relevant pH-values mimicking the neutral and slightly acidic parts of the intestine [14]. Using simple bulk-mixing, the complexes formed between insulin or PTH(1-34) and penetratin were examined with respect to size-distributions employing dynamic light scattering (DLS), association efficiency using ultracentrifugation, and folding propensity using circular dichroism (CD) spectroscopy. The effect of complexation on the potential of penetratin to interact with/disrupt lipid membranes was studied using fluorescence confocal microscopy. The chemical stability of insulin, PTH(1-34), and penetratin was assessed following apical incubation with the human colon adenocarcinoma Caco-2 cell culture model [15]. Finally, the penetratin-mediated transepithelial permeation of insulin and PTH(1-34) was evaluated in vitro using the Caco-2 cell culture model and in vivo following intra-intestinal injection in rats.

## 2. Materials and Methods

Human recombinant insulin (5807.6 g/mol, Zn-free) was provided by Sanofi (Frankfurt, Germany). Synthetic PTH(1-34) (4117.8 g/mol, purity > 95%) and penetratin (RQIKIWFQNRRMKWKK) (2246.7 g/mol, purity > 95%) were purchased from Bachem (Torrance, CA, USA) and Synpeptide (Shanghai, China), respectively. Thioflavin T (ThT) (T3516), Nile Red (N3013), and all other chemicals were obtained from Merck (Buchs, Switzerland) unless otherwise stated.

### 2.1. Preparation of Insulin-Penetratin and PTH(1-34)-Penetratin Samples

Using low-binding Eppendorf tubes, insulin, PTH(1-34), or penetratin was dissolved in ultrapure water (18.2 MΩ × cm) obtained from a Barnstead^TM^ water purification system (Thermo Scientific, Wilmington, NC, USA). For insulin, volumes of 0.1 M HCl were added until completely dissolved before neutralization by the addition of equal volumes 0.1 M NaOH. The insulin, PTH(1-34), and penetratin solutions were passed through a 0.22 µm Millex Millipore filter (MB Millipore, Billerica, MA, USA) before the concentrations were determined using a Nanodrop 2000c (Thermo Scientific, Wilmington, NC, USA). The insulin, PTH(1-34), and penetratin stock solutions were further diluted to relevant test concentrations in Hanks’ Balanced Salt Solution (HBSS) (Gibco, Invitrogen, Nærum, Denmark) added 10 mM 2-(N-morpholino)ethanesulfonic acid (MES) (mHBSS) (AppliChem, Darmstadt, Germany), and adjusted to pH 5 or 6.5, or 10 mM 4-(2-hydroxyethyl)-1- piperazineethanesulfonic acid (HEPES) (hHBSS) (AppliChem) and adjusted to pH 7.4.

### 2.2. Cell Culture Model

Caco-2 cells were purchased from the American Type Culture Collection (ATCC, Manassas, VA, USA) and grown in Dulbecco’s Modified Eagle Medium (DMEM) supplemented with 2 mM L-glutamine, 0.1 mM non-essential amino acids, 90 U/mL penicillin, 90 µg/mL streptomycin and 10% (*v/v*) fetal bovine serum (FBS) (Thermo Fischer Scientific, Slangerup, Denmark) at 37 °C and 5% CO_2_ in T175 cell culture flasks (Corning Costar, Costar, NY, USA). At 80% confluency the cells were sub-cultured by detaching them from the culture flasks by trypsin-EDTA treatment. For the permeation study 1 × 10^5^ cells were grown on polycarbonate filters with a diameter of 1.12 cm^2^ and a pore size of 0.4 µm placed in a 12-well Transwell^®^ plate (Corning Costar, Costar, NY, USA) for 20 days prior experimental use.

### 2.3. Size Distribution

Size distributions according to the distribution by volume were determined using a Zetasizer Nano ZSP (Malvern Instruments, Worcestershire, UK) equipped with a 633 nm laser at 25 °C with 10–20 runs of 10 s and N = 3. The size measurements were performed on 180 µM insulin or PTH(1-34) and 720 µM penetratin in mHBSS adjusted to pH 5 or 6.5 or in hHBSS adjusted to pH 7.4.

### 2.4. Association Efficiency

Samples containing 180 µM insulin or PTH(1-34) and 720 µM penetratin were subjected to ultracentrifugation (25,000× *g*, 30 min, 20 °C) using a 5417 R centrifuge equipped with a FA-45-24-11 rotor (Eppendorf, Hamburg, Germany). Insulin, PTH(1-34), and penetratin left in the supernatant was quantified by reverse phase high-pressure liquid chromatography (HPLC) using a Prominence HPLC system (Shimadzu, Kyoto, Japan) equipped with an Aeris PEPTIDE XB-C18 column (3.6 µm core shell particles; Torrance, CA, USA). A gradient elution from 20–70% mobile phase B (acetonitrile/ultrapure water 95:5 and 0.1% trifluoroacetic acid (TFA) (*v*/*v*)) in mobile phase A (ultrapure water/acetonitrile 95:5 and 0.1% TFA (*v*/*v*)) over 10 min was applied using a flow rate of 0.8 mL/min and an injection volume of 10 µL. UV-detection was performed at 218 nm.

### 2.5. Secondary Structure

CD spectra were recorded in the range of 180–260 nm with 0.5 nm steps on a Chirascan CD spectrometer (Applied Photophysics, Leatherhead, UK) using a 1 mm cuvette (Helma Analytics, Müllheim, Germany). Measurements were performed at 37 °C in 10 mM phosphate buffer (pH 5, 6.5, or 7.4) with 50 µM insulin or PTH(1-34) and 200 µM penetratin. All spectra represent an average of 3 scans which have been normalized to baseline and transformed into mean residue ellipticity (MRE):

MRE = (*MRW* × *Ө*)/(10 × *l* × *c*) (1)

where *MRW* is the mean residue weigh, *Ө* is the ellipticity, *l* is the path length, and *c* is the sample concentration.

### 2.6. Liposomes

For fluorescence confocal microscopy, anionic POPC:POPG (80:20 molar ratio) liposomes were produced by the thin film method as described by Foged et al. [16]. Briefly, dry lipid films were formed in round bottom flasks by applying vacuum over night before hydration in buffer (10 mM HEPES, 150 mM KCl, pH 7.4) with agitation every 10 min for 1 h before annealing for 1 h.

### 2.7. Visualization of Supramolecular Structures

Samples containing 50 µM insulin or PTH(1-34) and 200 µM penetratin were added 100 µM ThT and 100 µM Nile Red. 50 µL were placed into microscope slides and imaged at 1024 × 1024 pixel resolution using a Leica TCS SP5 confocal laser scanning microscope with a 63× oil objective NA = 1.4 (Leica Microsystems, Wetzlar, Germany) and a scanning frequency of 200 Hz. ThT fluorescence was measured under 458 nm excitation in the range 470–550 nm (green channel) and Nile Red fluorescence was measured under 543 excitation in the range 600–700 nm (red channel). In the two color experiments, images were sequentially acquired in order to avoid bleed through artifacts.

### 2.8. Degree of ThT and Nile Red Binding to Supramolecular Complexes

The fluorescence in samples containing 50 µM insulin or PTH(1-34), 200 µM penetratin and 100 µM ThT (ex 450/em 485) or 100 µM Nile Red (ex 552/em 636) were recorded in black clear-bottom 96-well over 3 h using a CLARIOstar plate reader (BMG Labtech, Offenburg, Germany).

### 2.9. In Vitro Stability

The stability of insulin, PTH(1-34), and penetratin was assessed during apical incubation in Caco-2 cell monolayers as previously described by Trehín et al. [17]. Briefly, The Caco-2 cell monolayers were washed twice apical and basolateral with 37 °C hHBSS and equilibrated to room temperature in the last washing volume. The transepithelial electrical resistance (TEER) was measured using an EVOM equipped with an Endohm-12 cup (World Precision Instruments, Sarasota, FL, USA) to validate the barrier integrity of the monolayer. Following equilibration to 37 °C the filter inserts were transferred to empty 6-well plates in order to block permeation across the monolayer. The buffer was removed from the apical compartment and the stability study was initiated by adding 800 µL of the prepared test samples. 50 µL samples were collected in HPLC vials prefilled with 5 µL 1% (*v*/*v*) formic acid in methanol at time-point 0, 30, 60, 90, 120, 150, 180, 210, and 240 min. The kinetics of the metabolic degradation was evaluated by analytical RP-HPLC as described under the ultracentrifugation procedure. Data for penetratin alone or in the presence of insulin or PTH(1-34) was analyzed according pseudo-first order kinetics and the half-lives (t_1/2_) calculated:

t_1/2_ = ln(2)/[d(logC)/dt] (2)

where C is the peptide concentration and t is the incubation time.

### 2.10. In Vitro Transepithelial Permeation

Caco-2 cell monolayers were washed twice apical and basolateral with 37 °C hHBSS. The cells were equilibrated to room temperature in the last washing volume before TEER was measured using an EVOM equipped with an Endohm-12 cup (World Precision Instruments, Sarasota, FL, USA) and equilibration to 37 °C. The buffer was removed from the basolateral and apical compartments and 500 µL test samples containing 5 µM insulin or PTH(1-34) and 20 µM penetratin was added to the apical chamber. The permeation study was initiated by careful transfer of the filter inserts to fresh 12-well plates containing 1 mL 37 °C hHBSS. The experiment was performed on a 37 °C shaking table (50 rpm).

100 µL test samples were withdrawn from the basolateral compartment at time-points 0, 60, 120 and 180 min and stored at −20 °C. The sample volume was immediately replaced with 37 °C hHBSS. Insulin and PTH(1-34) were quantified using an insulin ELISA kit (Mercodia, Uppsala, Sweden) or a PTH(1-34) EIA kit (Bachem, Torrance, CA, USA) according to the manufacturer’s directions with absorbance measurement at 450 nm employing a FLUOstar OPTIMA plate reader (BMG Labtech, Offenburg, Germany).

The apparent permeability coefficient (P_app_) was calculated:

P_app_ (cm/s) = (dQ/dt)/(A × C_0_) (3)

where dQ/dt is the steady state flux, A (1.12 cm^2^) is the area of the Caco-2 monolayer and C_0_ is the initial donor concentration.

### 2.11. Epithelial Cell Viability

The 3-(4,5-dimethyl-thiazol-2-yl)-5-(3-carboxymethoxyphenyl)-2-(4-sulfophenyl)-2H-tetrazolium (MTS)/phenazine methosulfate (PMS) assay (as previously described by Cory et al. [18]) was employed to determine the cellular viability of the Caco-2 cells after each permeation study. Briefly, 320 µL MTS/PMS solution containing 240 µg/mL MTS and 2.4 µg/mL PMS (Promega, Madison, WI, USA) was added the cell monolayers and incubated for 1.5 h with orbital shaking (50 rpm) at 37 °C. Two 100 µL samples were withdrawn from each well and the absorbance was measured at 492 nm on a FLUOstar OPTIMA plate reader (BMG Labtech, Offenburg, Germany).

The relative viability was calculated by using the equation:

Relative viability (%) = (A_sample_ − A_SDS_)/(A_buffer_ − A_SDS_) × 100% (4)

where A_sample_ is the absorbance of the test sample, A_SDS_ is the absorbance of the positive control (0.2% (*w/v*) sodium dodecyl sulphate (SDS)) corresponding to 0% cell viability, and A_buffer_ is the absorbance of the negative control corresponding to 100% cell viability.

### 2.12. In Situ Rat Intestinal Injection

Male Sprague Dawley rats (Taconic, Ejby, Denmark) weighing ~250 g were allowed to acclimatize for at least 8 days at 20–22 °C and 45–65% humidity with free access to water and food. The rats were fasted for 18 h prior to surgery. Hypnorm (fentanyl 0.315 mg/mL and fluanisone 10 mg/mL) (Glostrup apotek, Glostrup, Denmark) and Dormicum (midazolam 5 mg/mL) (Nomeco, Copenhagen, Denmark) were mixed 1:1 (*v*/*v*) with ultrapure water and used for anesthesia. The initial dose was 2 mL/kg and subsequent doses of 1 mL/kg were given every 20 min within the first hour and thereafter every 40 min to keep the animals anaesthetized. All doses of anesthetics were administered subcutaneously in the neck region. The abdominal wall was cut open and the small intestine localized. A 10 cm PE-50 catheter (Smiths Medical, Keene, NH, USA) was inserted into the middle of the intestine and secured with a 3/0 suture (Braun, Tuttlingen, Germany). Care was taken not to interrupt the mesenteric blood flow. The intestine was hydrated with 1–2 mL isotonic sodium chloride solution before the rat abdominal wall was closed with 9 mm clips using the AutoClip Wound Closure System (Alzet, Cupertino, CA, USA). The animal was left on a 37 °C heating plate (Scandidact, Kvistgaard, Denmark) for 30 min prior dosing of the test samples to allow normalization of the potentially elevated blood glucose level resulting from stress induced by handling and surgery. 500 µL test samples containing 180 µM insulin (50 IU/kg) or PTH(1-34) and 720 µM penetratin were injected into the catheter. A volume of 200 µL air was injected immediately after dosing to fully empty the catheter into the intestinal lumen. 200 µL blood samples were collected from the tail vein at time points 0, 5, 15, 30, 45, 60, 90, and 120 min into EDTA-coated microcuvette vials (Sarstedt, Nümbrecht, Germany). The blood samples were placed on ice prior isolation of the plasma by centrifugation at 2000× *g* at 4 °C for 10 min (Eppendorf centrifuge 5424, Eppendorf, Hamburg, Germany). Blood glucose was measured at time points of 15, 30, 45, 60, 90, and 120 min after dosing using an On-Call blood glucose meter (Acon, San Diego, CA, USA). After the final blood sampling, the animals were euthanized while still under anesthesia using 90% CO_2_ in O_2_ [19]. The abdominal wall of the rats was reopened after the experiment to ensure the catheter was still in place. Likewise, control of potential damage to the intestine was determined macroscopically. The experiments were conducted in strict compliance with the national animal research license 2009/561-1622.

### 2.13. Data and Statistical Analysis

Data processing was carried out using Microsoft Office Excel 2010 and GraphPad Prism version 6 (GraphPad Software, San Diego, CA, USA). Statistical analysis was done in GraphPad Prism using one-way analysis of variance (ANOVA) with Tukey’s multiple comparisons test. Data are presented as mean ± standard deviation (SD) or standard error of mean (SEM) with N representing the number of experimental replicates and n representing number of biological replicates.

## 3. Results

### 3.1. Complex Size is Influenced by the Isoelectric Point of the Therapeutic Peptide

**Figure 1:**
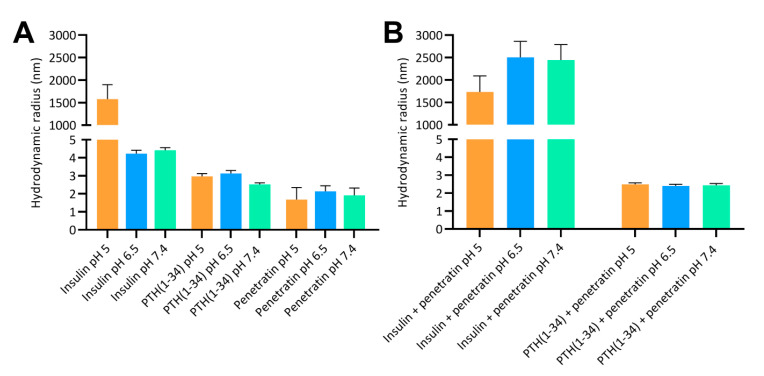
Hydrodynamic radii according to volume distributions at pH 5, 6.5, and 7.4 determined by dynamic light scattering. (**A**): 180 µM insulin, 180 µM PTH(1-34), or 720 µM penetratin. (**B**): 180 µM insulin or 180 µM PTH(1-34) in physical mixture with 720 µM penetratin. Data are presented as volume mean sizes (N = 3, mean ± SD).

The resulting complex sizes obtained after mixing insulin or PTH(1-34) with penetratin were studied using a 1:4 insulin or PTH(1-34) to penetratin molar mixing ratio, as this ratio previously showed efficient penetratin-mediated intestinal insulin delivery [6,11]. The mean sizes of the formed complexes were assessed by DLS and dominant sizes according to volume distributions at pH 5, 6.5, and 7.4 are shown in Figure 1. In samples containing only insulin, self-association into large complexes with mean volume sizes of 1579 ± 319 nm were observed at pH 5 (Figure 1A). In contrast, larger complexes resulting from self-association did not dominate in the insulin samples at pH 6.5 or 7.4 or in samples containing only PTH(1-34) or penetratin at pH 5, 6.5, or 7.4. Mixing insulin with penetratin at pH 5 resulted in complexes with comparable sizes as observed for insulin alone (Figure 1B). Larger insulin-penetratin complexes were detected at pH 6.5 (2503 ± 360 nm) and pH 7.4 (2443 ± 348 nm) when compared to pH 5 (1732 ± 359 nm). The sizes determined in the PTH(1-34)-penetratin samples were comparable to those determined in the samples containing only PTH(1-34) or penetratin, with volume mean sizes assessed to 2.5 ± 0.1 nm at pH 5, 2.4 ± 0.1 nm at pH 6.5, and 2.4 ± 0.1 nm at 7.4 (Figure 1B). All penetratin and PTH(1-34)-penetratin samples contained a minor fraction of supramolecular complexes as observed upon evaluation of the size distributions by intensity (Figure S1). In addition, even larger complexes may be present, but not detectable using DLS which is not applicable for detection of structures exceeding 10 µm.

### 3.2. Insulin and PTH(1-34) Association Efficiency with Penetratin Differs according to pH and Therapeutic Cargo

**Figure 2:**
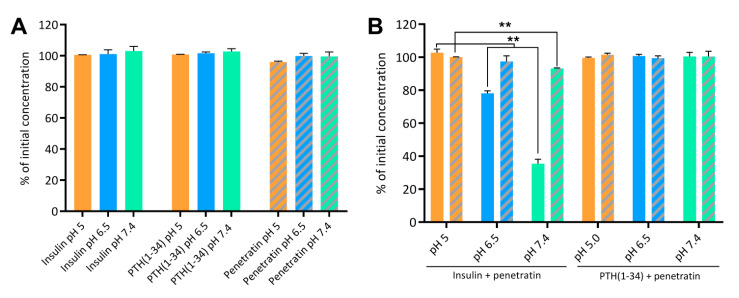
Insulin, PTH(1-34), and penetratin left in the supernatant following ultracentrifugation alone (**A**) or as physical mixtures of insulin and penetratin or PTH(1-34) and penetratin (**B**) at pH 5, 6.5, or 7.4. Full columns represent insulin or PTH(1-34) and dashed columns represent penetratin. Data are presented as % of initial concentration (N = 2, mean ± SD). Level of significance is **: *p* < 0.01 (unpaired *t*-test).

To investigate the association efficiency between insulin or PTH(1-34) and penetratin at pH 5, 6.5, and 7,4, the samples were subjected to ultracentrifugation with subsequent detection of free insulin, PTH(1-34), and penetratin in the supernatant (Figure 2). No insulin, PTH(1-34), or penetratin prepared individually at pH 5, 6.5, or 7.4 were lost to sedimentation of larger complexes formed as a result of self-association (Figure 2A). However, subjecting the insulin-penetratin samples to ultracentrifugation resulted in significant loss of insulin from the pH 6.5 (21.8 ± 1.5%) and pH 7.4 (64.5 ± 2.7%) samples as well as a minor, but still significant loss of penetratin in the pH 7.4 sample (6.8 ± 0.4%) (Figure 2B). Neither PTH(1-34) nor penetratin were lost to sedimentation of larger complexes following ultracentrifugation of the PTH(1-34)-penetratin samples at pH 5, 6.5, or 7.4 (Figure 2B).

### 3.3. Complexation at pH 6.5 and 7.4 Affects the Secondary Structure in the Insulin-Penetratin Samples

**Figure 3:**
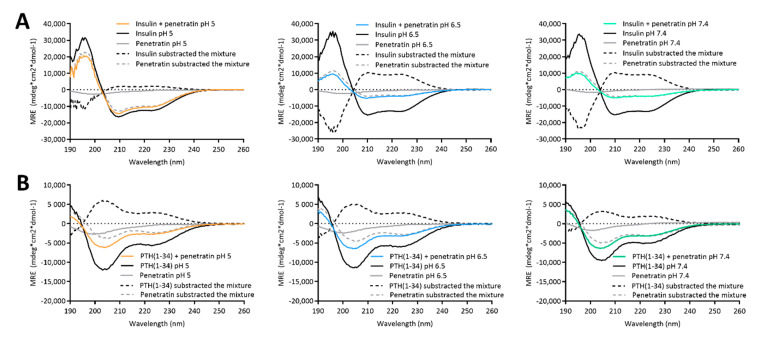
Circular dichroism spectra resulting from mixing 50 µM insulin (**A**) or PTH(1-34) (**B**) with 200 µM penetratin at pH 5, 6.5, or 7.4. Dashed lines represent spectra resulting from subtracting the signal from samples containing only insulin/PTH(1-34) or penetratin from the signal originating from samples containing insulin or PTH(1-34) in mixture with penetratin.

The biologically active forms of insulin [20] and PTH(1-34) [21] adapt well-defined secondary structures. In addition, the ability of penetratin to adapt into an alpha helical conformation in the vicinity of lipid membranes [3,22] may be important for its membrane permeating potential [23,24]. Therefore, the effect of complexation on the secondary folding propensity was studied using CD spectroscopy (Figure 3). Alone in solution, the secondary structure of insulin, PTH(1-34), and penetratin was not affected by the sample pH (Figure S2). Insulin (Figure S2A) and PTH(1-34) (Figure S2B) adapted into well-defined α-helical structures as evident by the characteristic minima and maximum observed at 208/222 nm and 193 nm, respectively. Penetratin alone did not adapt into a well-defined secondary structure (Figure S2C).

The CD spectra obtained from samples containing mixtures of insulin or PTH(1-34) and penetratin all revealed α-helical content (Figure 3). The spectrum resulting from mixing insulin with penetratin at pH 5 was similar to the spectrum obtained for insulin alone, whereas the spectra obtained from mixing insulin with penetratin at pH 6.5 and 7.4 revealed less α-helical content when compared to insulin alone (Figure 3A). Subtraction of the penetratin spectra from the insulin-penetratin spectra resulted in profiles similar to the spectra originating from the insulin-penetratin mixtures (Figure 3A). In the PTH(1-34)-penetratin samples, subtraction of the penetratin signal resulted in a similar α-helical profile as obtained from the mixture, though slightly shifted upwards (Figure 3B). On the contrary, the subtraction of the insulin or PTH(1-34) spectra from spectra obtained from insulin- or PTH(1-34)-penetratin mixtures resulted in profiles differing from the spectra recorded from both insulin, PTH(1-34), and penetratin alone as well as spectra from the mixtures containing insulin or PTH(1-34) and penetratin.

### 3.4. Membrane Activity of Penetratin Is Maintained in the Presence of Insulin or PTH(1-34) and Is Independent of pH

**Figure 4:**
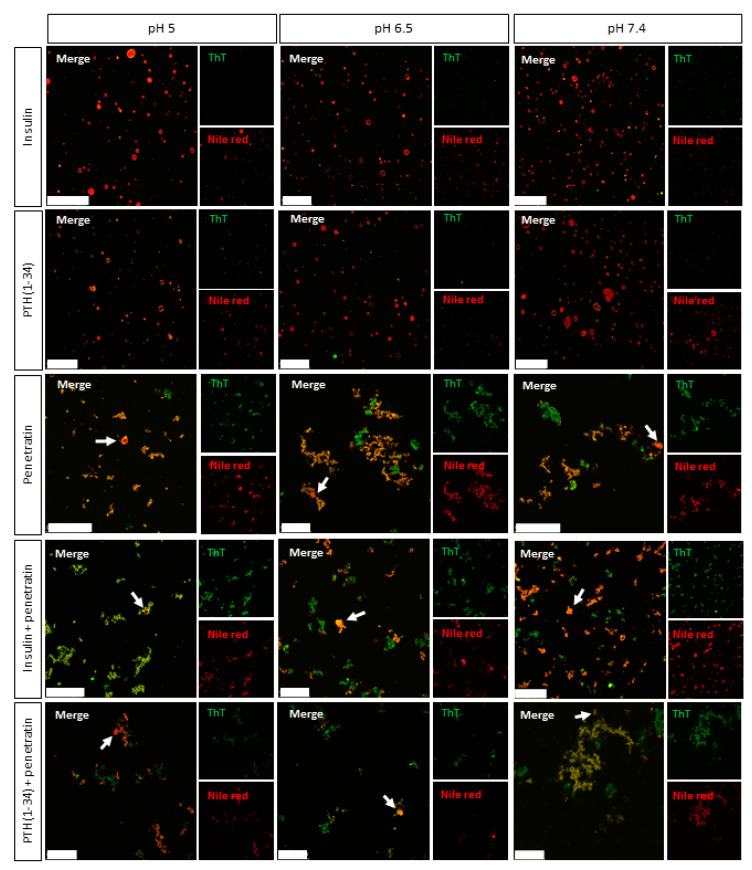
Representative confocal microscopy images of complexes obtained from mixing 50 µM insulin or PTH(1-34) with 200 µM penetratin at pH 5, 6.5, or 7.4 in the presence of POPC:POPG (80:20 molar ratio) liposomes and Thioflavin T (ThT) (green) and Nile Red (red). Arrow heads: Examples of unruptured liposomes in samples containing penetratin. Scale bars: 50 µm.

The ability of penetratin to interact with/disrupt liposomal structures, alone or in mixture with insulin or PTH(1-34), were studied using fluorescence confocal microscopy (Figure 4). POPC:POPG liposomes were included as membrane mimics. ThT and Nile Red, having affinity for amyloid fibrils and hydrophobic patches, respectively [25,26], were included for visualization of self-associated molecules, insulin/PTH(1-34)-penetratin complexes, and lipid structures.

Without liposomes, some Nile Red fluorescence was detected in the pH 5 insulin sample (Figure S3), whereas no significant Nile Red fluorescence was observed in the pH 6.5 and 7.4 insulin samples or in any of the samples containing PTH(1-34) alone. Neither of the samples with insulin or PTH(1-34) alone stained positive for ThT. In the penetratin samples, few structures were observed at pH 5, whereas multiple supramolecular structures were present at pH 6.5 and 7.4. The smaller structures measured by DLS at nanometer scale of e.g., the PTH(1-34)-penetratin samples (Figure 1) were below instrumental resolution for confocal microscopy. Moreover, these nanometric structures may not interact with ThT and/or Nile Red, which is essential for detection using the confocal microscopy setup. The two methods allow detecting structures in two different spatial ranges. Fluorescence microscopy methods mainly allow characterization of micron scale objects in the sample, whereas DLS is only suitable for detection of particle sizes in the nanometer to low-micrometer range. To gain further insight into potential pH-dependent changes on complexation between insulin or PTH(1-34) and penetratin, ThT and Nile Red fluorescence was analyzed upon incubation in a 96-well plate (Figure S4). An increase in ThT fluorescence above background was observed for insulin at all pH-values tested, whereas no pronounced increase in ThT fluorescence above background was observed in the PTH(1-34) or in the penetratin samples (Figure S4A). On the other hand, mixing insulin with penetratin at pH 7.4 gave a 4.5-fold increase in ThT fluorescence above background. A 2-fold increase in ThT fluorescence above background was observed upon mixing PTH(1-34) with penetratin at pH 5, 6.5, and 7.4. No Nile Red fluorescence different from the background fluorescence was observed in either of the samples containing insulin, PTH(1-34), or penetratin alone, nor in samples containing insulin or PTH(1-34) mixed with penetratin (Figure S4B). The dye fluorescence intensities of ThT or Nile Red did not differ among the pH-values included (Figure S4C).

Upon the addition of liposomes, no supramolecular structures were observed in samples containing solely insulin or PTH(1-34) (Figure 4). It was, however, not possible to distinguish potential self-associated insulin stained with Nile Red at pH 5 (Figure S3) from the Nile Red stained liposomes (Figure 4). A number of supramolecular structures were observed in the penetratin samples as well as in mixtures of insulin or PTH(1-34) and penetratin. The amount of detectable intact liposomal structures was low in all samples containing penetratin (arrowheads Figure 4) when compared to samples containing solely insulin or PTH(1-34).

### 3.5. The Stability of Penetratin during Incubation with Caco-2 Cell Monolayers Is Increased by Lowering the Sample pH

To assess whether complexation may be beneficial in terms of hindering enzymatic degradation of insulin, PTH(1-34), and/or the carrier peptide penetratin, their proteolytic stability was evaluated during apical incubation on polarized Caco-2 cell monolayers (Table 1). Insulin remained stable during incubation for 4 h on the cell monolayers (Figure S5A). When applied in physical mixture with penetratin, insulin remained stable at pH 7.4 after 4 h incubation, whereas 89.9 ± 11.2% and 77.8.5 ± 3.6% of the initially applied insulin was detected after 4 h at pH 5 and pH 6.5, respectively (Figure S5B). PTH(1-34) appeared to be slightly less stable than insulin with 66.6 ± 8.5%, 71.7 ± 7.1%, and 73.6 ± 9.1% of intact PTH(1-34) remaining following 4 h incubation at pH 5, 6.5, and 7.4, respectively (Figure S5C). In the presence of penetratin, 72.4 ± 3.0%, 67.0 ± 2.6%, and 69.9 ± 2.7% of PTH(1-34) remained intact after 4 h at pH 5, 6.5, and 7.4, respectively (Figure S5D). No trend in pH-dependency with respect to neither insulin nor PTH(1-34) stability during incubation on the Caco-2 cell monolayers was observed. Penetratin was more prone to degradation than insulin and PTH(1-34) (Figure S6A–C) as also reflected in the calculated half-lives without or with the presence of insulin or PTH(1-34) (Table 1). Interestingly, the penetratin stability was highly pH-dependent, with longer half-lives obtained at pH 5 (160.2 ± 14.3 min) when compared to pH 6.5 (64.9 ± 14.0 min) and 7.4 (43.4 ± 11.0 min). In addition, even longer half-lives of penetratin were obtained as a result of mixing penetratin with insulin or PTH(1-34) at pH 5 and with insulin at pH 7.4, when compared to applying penetratin alone to the Caco-2 monolayers.

### 3.6. The Penetratin-Mediated Transepithelial Permeation of Insulin and PTH(1-34) is pH-Dependent

**Figure 5:**
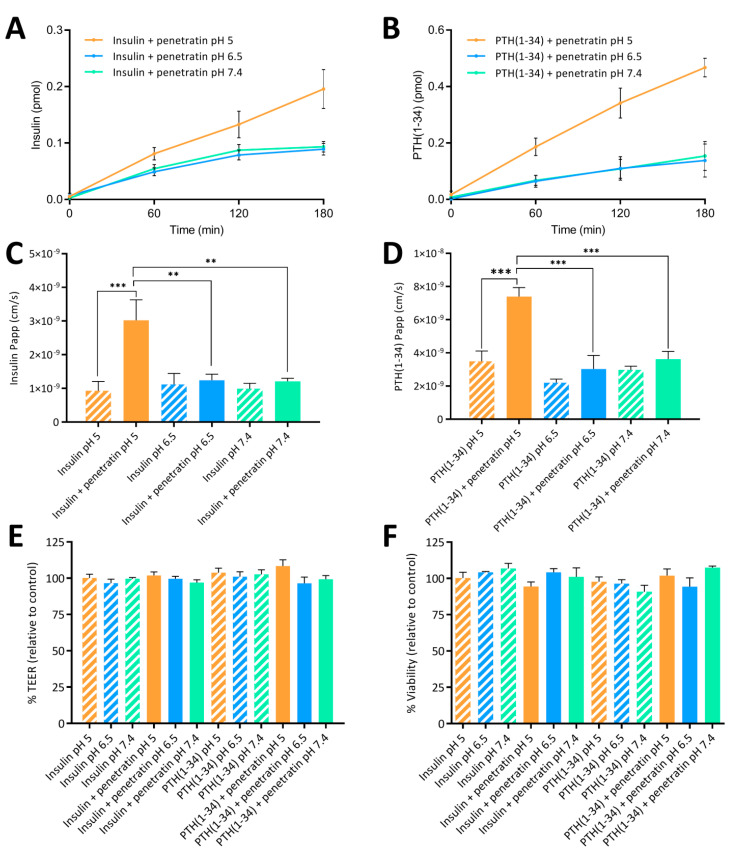
Permeation of insulin (**A**) or PTH(1-34) (**B**) across Caco-2 monolayers following application of 5 µM insulin or 5 µM PTH(1-34) in physical mixture with 20 µM penetratin at pH 5, 6.5, or 7.4 for 3 h. The apparent permeability coefficient (Papp) determined after application of 5 µM insulin (**C**) or PTH(1-34) (**D**) administered alone or in physical mixture with 20 µM penetratin at pH 5, 6.5, or 7.4. Apparent permeability (Papp) calculations are based on data from all time-points. TEER (**E**) and cell viability (**F**) evaluated following application of insulin or PTH(1-34) alone or in physical mixture with 20 µM penetratin at pH 5, 6.5, or 7.4 (N = 3, n = 3, mean ± SEM). Levels of significance are **: *p* < 0.01 and ***: *p* < 0.001 (one-way ANOVA with Tukey’s multiple comparisons test).

The effect of complex formation with respect to the ability of penetratin to mediate transepithelial permeation of insulin and PTH(1-34) was assessed at pH 5, 6.5, and 7.4 using polarized Caco-2 cell monolayers (Figure 5). At pH 6.5 and 7.4 no significant increase in the penetratin-mediated permeation of neither insulin (Figure 5A) nor PTH(1-34) (Figure 5B) was observed. Lowering the sample pH to 5, however, resulted in an increase in the transepithelial penetratin-mediated insulin (Figure 5A) and PTH(1-34) (Figure 5B) permeation as also reflected in the calculated apparent permeability values (Figure 5C,D). Lowering the sample pH from 6.5 or 7.4 to pH 5 did not increase neither insulin (Figure 5C) nor PTH(1-34) (Figure 5D) permeation when applied without penetratin to the Caco-2 cell monolayers. The peptide test concentrations were kept at 5 µM insulin or PTH(1-34) and 20 µM penetratin to exclude influence of potential adverse effects on barrier integrity (Figure 5E) and/or cell viability (Figure 5F), which would contribute to the observed transepithelial penetratin-mediated insulin and PTH(1-34) permeation (Figure 5A–D).

### 3.7. Penetratin Mediates pH-Dependent Permeation of Insulin and PTH(1-34) across Rat Intestinal Mucosa

**Figure 6:**
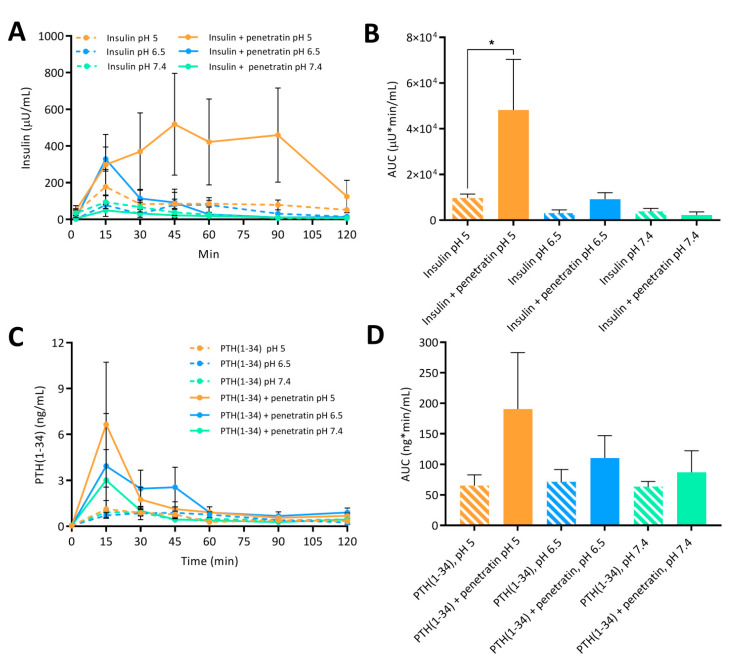
Plasma insulin (**A**) with associated AUC (**B**) and plasma PTH(1-34) (**C**) with associated AUC (**D**) following intraintestinal administration of insulin (50 IU/kg) or PTH(1-34) (1.2 mg/kg) alone or in physical mixture with penetratin corresponding to a molar 1:4 ratio at pH 5, 6.5, or 7.4 (N = 6, mean ± SD). Level of significance is *: *p* < 0.5 (one-way ANOVA with Tukey’s multiple comparisons test).

**Figure 7:**
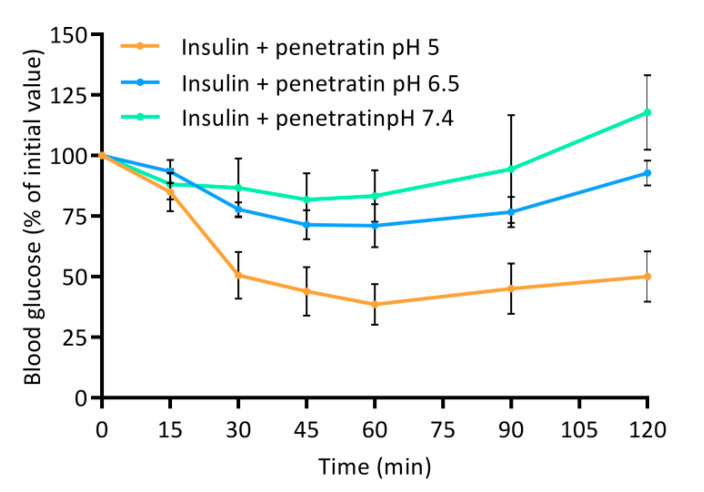
Blood glucose level depicted as % of initial value following intraintestinal administration of insulin (50 IU/kg) in physical mixture with 720 µM penetratin corresponding to a molar 1:4 ratio at pH 5, 6.5, or 7.4. Data are presented as % of initial value (N = 6, mean ± SD).

Finally, the documented potential of penetratin to facilitate transepithelial insulin and PTH(1-34) permeation in vitro (Figure 5A–D), was assessed in vivo by intraintestinal administration to rats (Figure 6). Moreover, the effect on blood glucose lowering was evaluated in parallel (Figure 7).

The greatest effect of co-administering insulin with penetratin on the resulting plasma insulin concentration was observed at pH 5 when compared to pH 6.5 and 7.4 (Figure 6A). This tendency was also reflected in the calculated AUC (Figure 6B). Moreover, a slight increase in the penetratin-mediated insulin delivery was observed at pH 6.5, whereas the plasma insulin concentration following co-administration with penetratin at pH 7.4 did not differ from administration of insulin alone at pH 7.4 (Figure 6A,B). Similarly, a pH-dependent trend for the penetratin-mediated PTH(1-34) delivery (pH 5 > pH 6.5 > pH 7.4) was reflected in the obtained C_max_ of plasma PTH(1-34) (Figure 6C) and the calculated AUC (Figure 6D). Following administration of PTH(1-34) alone, a fraction was detected in plasma, but no pH-dependency was observed. Finally, the pH-dependent effect observed on the penetratin-mediated insulin delivery across the rat intestinal mucosa (Figure 6A,B) was reflected in the blood glucose levels (Figure 7). At pH 5 a decrease to 38.5 ± 23.8% of the initial blood glucose level was observed at the 60 min time-point. Slight effects on the blood glucose levels were also observed following co-administration of insulin with penetratin at pH 6.5 and 7.4, reaching 71.1 ± 17.8% and 83.3 ± 21.2% of the initial blood glucose levels after 60 min, respectively. A slight increase in the blood glucose level was observed after the 90 min time-point. Especially for the pH 6.5 and 7.4 samples when compared to the 60 min time-point, thus demonstrating pH-dependent reversibility of the drop in blood glucose level. Administration of insulin alone at pH 5 and 6.5 was associated with a slight decrease to 86.9 ± 6.0% and 88.9 ± 11.1% of the initial blood-glucose level, respectively (Figure S7). Administration of insulin alone at pH 7.4 or penetratin alone at pH 5 did not affect the blood glucose level (Figure S7).

## 4. Discussion

In the present study the effect of sample pH was applied in the study of complexes formed between the therapeutic peptides insulin or PTH(1-34) and penetratin applied as a carrier peptide to enhance intestinal delivery. Focus was on studying the properties of the self-associated complexes, structure, membrane interaction, and stability to gain knowledge on mode of action and parameters important for successful penetratin-mediated transepithelial delivery of co-administered insulin and PTH(1-34).

Insulin self-association was observed at pH 5 using DLS and confocal- microscopy; likely due to the fact that pH 5 is close to the isoelectric point (5.3) of monomeric insulin [27]. Neither insulin at pH 6.5 or 7.4 nor PTH(1-34) appeared to self-associate to the same extent. Some penetratin self-association was observed at all pH-values; similar to previously reported for penetratin [28], Tat, nona-arginine [29] and other membrane translocating peptides [30]. Supramolecular µm-sized insulin-penetratin complexes dominated the samples at pH 6.5 and 7.4, which is in agreement with earlier studies, documenting supramolecular complexes as a result of mixing insulin with penetratin or analogues thereof [11] or with octaa-arginine [31]. The size distribution for the pH 5 insulin-penetratin sample did not differ from the pH 5 insulin sample, thereby indicating that the insulin-penetratin complexation is more pronounced at pH 6.5 and 7.4 as compared to pH 5. The pI of insulin and PTH(1-34) is 5.3 and 8.3, respectively, and the pI of penetratin is 12.3 leaving it positively charged at pH 5, 6.5, and 7.4. Thus, electrostatic interactions appear to be main determinants for the observed complex formation within the insulin-penetratin samples. The association efficiency between insulin or PTH(1-34) and penetratin was indirectly quantitated by subjecting the samples to ultracentrifugation. Insulin sedimentation was observed in samples of pH 6.5 and 7.4; thereby supporting the earlier observation of larger complexes resulting from mixing insulin with penetratin. In line with this result, an earlier study investigated the binding strength between insulin and penetratin at pH 5, 6, and 7, demonstrating limited interaction between insulin and penetratin at pH 5, whereas the strongest interactions were observed at pH 6 and 7 [32]. Almost no sedimentation of penetratin was observed in the ultracentrifugated pH 6.5 and 7.4 insulin-penetratin samples; thereby pointing to that insulin may be the dominant molecule present in the insulin-penetratin complexes. Though penetratin appears to self-associate, no sedimentation was observed. Similarly, neither PTH(1-34) nor penetratin were lost to sedimentation following ultracentrifugation alone or in mixture; despite some complexation was observed using DLS and confocal microscopy. We ascribe this discrepancy to that the observed complexes in those samples may have been only loosely associated complexes that de-assembled during the centrifugation step or were of such small size and density that they could not be efficiently spun down.

Effects on the secondary structure content was studied in the physical mixtures containing insulin or PTH(1-34) and penetratin using CD spectroscopy. The secondary structure in the insulin-penetratin sample was highly pH-dependent as evident by the minor amount of α-helical content in the pH 6.5 and 7.4 samples as compared to the pH 5 sample. Subtracting the penetratin spectra from the insulin-penetratin samples did not affect the resulting profile, thus implying little impact from penetratin on the overall secondary structure content in the mixtures. The more pronounced interaction between insulin and penetratin in the pH 6.5 and 7.4 samples may therefore affect the ability of insulin to adapt an α-helical conformation.

No pH-dependence was observed in the resulting secondary structure within the PTH(1-34)-penetratin samples, which is well in line with the lack of efficient association between PTH(1-34) and penetratin at the pH-values included in the present study. Thus, the ability to form large complexes with strong intermolecular interactions appears to be associated with some structural changes at the level of secondary structure as illustrated for the pH 6.5 and 7.4 insulin-penetratin samples.

The effect of mixing penetratin with insulin or PTH(1-34) with respect to interaction with lipid membranes was studied using fluorescence confocal microscopy. ThT and Nile Red were included as hydrophobic domain dyes to allow detection of the complexes and the liposomal structures. Neither Nile Red- nor ThT-staining was observed in the pH 6.5 or 7.4 insulin samples or in any of the PTH(1-34) samples, which in line with the DLS measurements, indicates absence of larger complexes. It should be noted, though, that confocal microscopy and DLS should be considered complementary acknowledging that they are applied to investigate different assembly size scales. All penetratin samples as well as samples containing mixtures of penetratin and insulin or PTH(1-34) stained positive for both dyes. The morphology of the observed aggregates and complexes appeared similar to the earlier observed so-called crinkled aggregates of concanavalin A staining positive for ThT [33]. At the applied peptide to lipid ratios, the addition of liposomes revealed penetratin-mediated rupture of the majority of the liposomal structures both without and with the presence of insulin or PTH(1-34), and without any obvious pH-dependence. Thus, penetratin appears to exert its effect on lipid membranes even as self-associated or in strong association with its therapeutic cargo, as observed for the pH 6.5 and 7.4 insulin-penetratin samples. In addition to the disappearance of the liposomal structures, some fusion and/or aggregation of the liposomal structures was detected (Figure S8); similar to recently observed as a result of the application of nona-arginine to giant unilamellar vesicles (GUVs) [34] or various cationic CPPs to POPC:POPG liposomes [28]. Liposomes do, however, not reflect the complexity of the intestinal epithelium, which likely explain the lack of observed penetratin-mediated transepithelial transport at pH 6.5 and 7.4 in vitro and limited effect in vivo.

Peptides and proteins are inherently prone to degradation when presented to the metabolic barrier of an epithelium [35,36]. Therefore, the enzymatic stability of penetratin is of great importance for its ability to mediate transepithelial permeation of co-administered cargo drugs such as insulin and PTH(1-34). The co-administration approach may positively influence the stability of penetratin when conditions favor complex formation, thereby retaining the cell-penetrating propensity of penetratin for a longer time-period. On the other hand, degradation of penetratin may have beneficial effects in terms of releasing the therapeutic cargo as well as the physiological clearance of penetratin. In the present study, the penetratin, insulin, and PTH(1-34) resistance towards enzymatic degradation was assessed during apical incubation on polarized Caco-2 cell monolayers. Penetratin was less stable when compared to insulin and PTH(1-34); and that in a pH-dependent manner, with longer half-lives obtained at pH 5 than at pH 6.5 and 7.4. In addition, the complexation with insulin at pH 7.4 appears to positively influence the penetratin stability; which was, however, not the case at pH 6.5. Thus, the better association efficiency observed in the pH 7.4 insulin-penetratin sample, likely adds to the longer half-life observed at pH 7.4 but not at pH 6.5, when compared to applying penetratin alone or together with PTH(1-34) at pH 6.5 or 7.4.

Finally, the ability of penetratin to facilitate transepithelial insulin and PTH(1-34) permeation was assessed in vitro using polarized Caco-2 cell monolayers and in vivo following intestinal injection in rats. A pH-dependent penetratin-mediated transepithelial permeation of insulin and PTH(1-34) was observed both in vitro and in vivo. In addition, the increase in the penetratin-mediated insulin delivery observed at pH 5 when compared to pH 6.5 and 7.4 was associated with a decrease in the blood glucose level. Thus, the formation of complexes observed in the insulin-penetratin samples at pH 6.5 and 7.4, which displayed stronger intermolecular interactions than at pH 5, did not have a positive impact on the transepithelial insulin permeation. In contrast, mixing insulin with penetratin at pH 6.5 and 7.4 resulted in visible precipitates in the concentrations applied in vivo (Figure S9). Visible precipitation was not evident for PTH(1-34) when mixed with penetratin, which is in line with the results from DLS and the ultracentrifugation experiments. Despite that, some effect of co-administering insulin with penetratin was observed at pH 6.5 with respect to plasma insulin and an associated decrease in blood glucose level. This observation corresponds well with an earlier study, demonstrating dissociation of insulin-penetratin aggregates following addition to isolated rat intestinal fluid [13]. Poor association between insulin and penetratin was observed at pH 5, thereby indicating that the ability of penetratin as a carrier for transmucosal insulin delivery is impaired by high association efficiencies resulting from physical mixing of molecules with distinctly opposing net charges. However, completely avoiding the formation of complexes appears not to be a general solution to increase the penetratin-mediated transepithelial permeation of a co-administered cargo. No or little penetratin-mediated PTH(1-34) permeation was observed at pH 6.5 and 7.4 in vitro and in vivo, respectively; and that despite poor association efficiency between PTH(1-34) and penetratin. Importantly, the increased penetratin stability obtained when lowering the sample pH from 7.4 or 6.5 to pH 5, may be the determining factor for the significant increase in insulin and PTH(1-34) oral delivery in vitro and in vivo.

## 5. Conclusions

The present work paves the way for further understanding the effect of formulation pH and complex formation when simply bulk-mixing insulin or PTH(1-34) with penetratin in order to obtain better transepithelial permeation of the cargo drug. The intermolecular association efficiency within complexes resulting from mixing insulin or PTH(1-34) with penetratin was pronounced at pH-values favoring electrostatic interactions, i.e., within the insulin-penetratin samples at pH 6.5 and 7.4. The formation of complexes in the insulin-penetratin samples observed at pH 6.5 and 7.4 resulted in less α-helical structures when compared to the pH 5 sample but did not affect the potential of penetratin to disrupt liposomal structures. The ability of penetratin to facilitate transepithelial permeation of insulin and PTH(1-34) in vitro and in vivo was, however, clearly favored at pH 5 when compared to pH 6.5 and 7.4. The penetratin stability was moreover positively affected by lowering the sample pH from 7.4 or 6.5 to pH 5. Thus, the better penetratin stability observed at pH 5 when compared to pH 6.5 and 7.4, rather than strong intermolecular interactions, may be the critical parameter in order to obtain successful transepithelial penetratin-mediated insulin and PTH(1-34) permeation.

The outcome of the present study implies that co-administration with penetratin may be a viable strategy to obtain intestinal delivery of therapeutic peptides, but control of the sample pH appears to be of utmost importance for future successful use.

## Figures and Tables

**Table 1:** Penetratin half-life determined after incubation of 25 µM penetratin or 20 µM penetratin co-administered with 5 µM insulin or PTH(1-34) on the apical side of Caco-2 monolayers for 4 h (N = 3, mean ± SD).

	Penetratin Half-Life (min)		
Sample	pH 5	pH 6.5	pH 7.4
Penetratin	160.2 ± 16.5 *^,a^	65.3 ± 5.2	44.4 ± 6.9
Insulin + penetratin	282.3 ± 17.9 ***^,b^	59.9 ± 14.1	77.7 ± 3.3 *^,d^
PTH(1-34) + penetratin	245.4 ± 12.3 ***^,c^	58.7 ± 5.8	54.2 ± 2.6

The values represent mean ± SD. Levels of significance are *: *p* < 0.05 and ***: *p* < 0.001 (paired *t*-test). ^a^ Compared to penetratin alone at pH 6.5 and 7.4; ^b/c^ compared to penetratin alone at pH 5; ^d^ compared to the penetratin alone at pH 7.4.

## Supplementary Materials

The following are available online at https://www.mdpi.com/1999-4923/12/10/993/s1, Figure S1: Representative size distributions by intensity of samples containing 720 µM penetratin (A–C) or 180 µM PTH and 720 µM penetratin (1-34) (D–E) at pH 5, 6.5, or 7.4 determined by dynamic light scattering, Figure S2: Circular dichroism spectra of 50 µM insulin (A), PTH(1-34) (B), and 200 µM penetratin (C) at pH 5, 6.5, or 7.4, Figure S3: Confocal and two-photon excitation microscopy images of complexes obtained as a result of mixing 50 µM insulin or PTH(1-34) with 200 µM penetratin at pH 5, 6.5, or 7.4 in the presence of Thioflavin T (ThT) (green) and Nile Red (red). Scale bars: 50 µm, Figure S4: Thioflavin T (ThT) (A) and Nile Red (B) increase over ThT or Nile Red background fluorescence (C) of samples containing 50 µM insulin or PTH(1-34) or 200 µM penetratin alone or as insulin/PTH(1-34) + penetratin mixtures prepared at pH 5, 6.5, or 7.4. (N = 3, mean ± SD), Figure S5: Stability of 5 µM insulin (A), 5 µM insulin in the presence of 20 µM penetratin (B), 5 µM PTH(1-34) (C), or 5 µM PTH(1-34) in the presence of 20 µM penetratin (D) during apical incubation on Caco-2 cell monolayers at pH 5, 6.5, or 7.4 over 4 h. Data are presented as % of initial concentration (N = 3, mean ± SD), Figure S6: Stability of 20 µM penetratin (A), 20 µM penetratin in the presence of 5 µM insulin (B), or 20 µM penetratin in the presence of 5 µM PTH(1-34) (C) during apical incubation with Caco-2 cell monolayers at pH 5, 6.5, or 7.4 over 4 h. Data are presented as % of initial concentration ± SD (N = 3, mean ± SD), Figure S7: Blood glucose following intraintestinal administration of insulin (50 IU/kg) at pH 5, 6.5, or 7.4 or 720 µM penetratin at pH 5. Data are presented as % of initial value (N = 6, mean ± SD), Figure S8: Confocal and two-photon excitation microscopy images of complexes obtained as a result of mixing 50 µM insulin with 200 µM penetratin at pH 6.5 in the presence of POPC:POPG (80:20 molar ratio) liposomes with addition of Thioflavin T (ThT) (green) and Nile Red (red), Figure S9: Visual inspection of pH 6.5 (left) and pH 7.4 (right) samples containing 180 µM insulin in physical mixture with 720 µM penetratin prior intraintestinal administration in rats.
