# Supplementary material for: Increased Carrier Peptide Stability through pH Adjustment Improves Insulin and PTH(1-34) Delivery In Vitro and In Vivo Rather than by Enforced Carrier Peptide-Cargo Complexation
Source: Pharmaceutics. 2020 Oct 20;12(10):993. doi: 10.3390/pharmaceutics12100993 (PMC7589992; doi:10.3390/pharmaceutics12100993)
Supplement: Supplementary file 1 [file pharmaceutics-12-00993-s001.pdf]

pharmaceutics

MDPI

Supplementary Materials

Increased Carrier Peptide Stability Through pH Adjustment Improves Insulin and PTH(1-34) Delivery In Vitro And In Vivo Rather than by Enforced Penetratin-Cargo Complexation

Mie Kristensen[1], Ragna Guldsmed Diedrichsen[1,2], Valeria Vetri[3], Vito Foderà[1,2] and Hanne Mørck Nielsen[1,2]*_

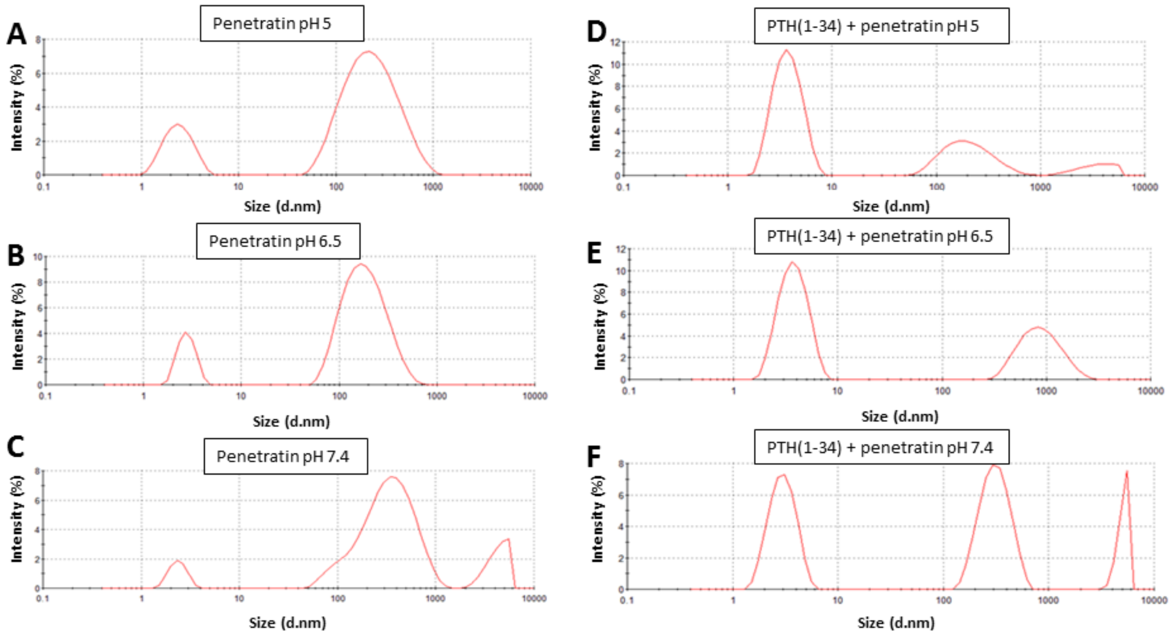

Figure S1. Representative size distributions by intensity of samples containing 720 µM penetratin (**A**-**C**) or 180 µM PTH and 720 µM penetratin (1-34) (**D**-**E**) at pH 5, 6.5, or 7.4 determined by dynamic light scattering.

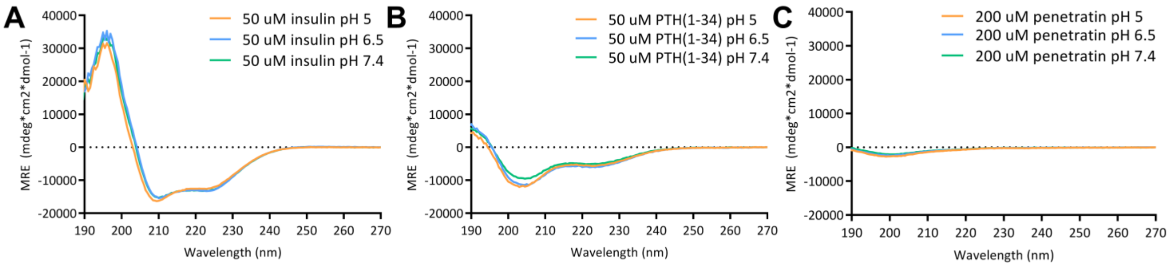

Figure S2. Circular dichroism spectra of 50 µM insulin (**A**), PTH(1-34) (**B**), and 200 µM penetratin (**C**) at pH 5, 6.5, or 7.4.

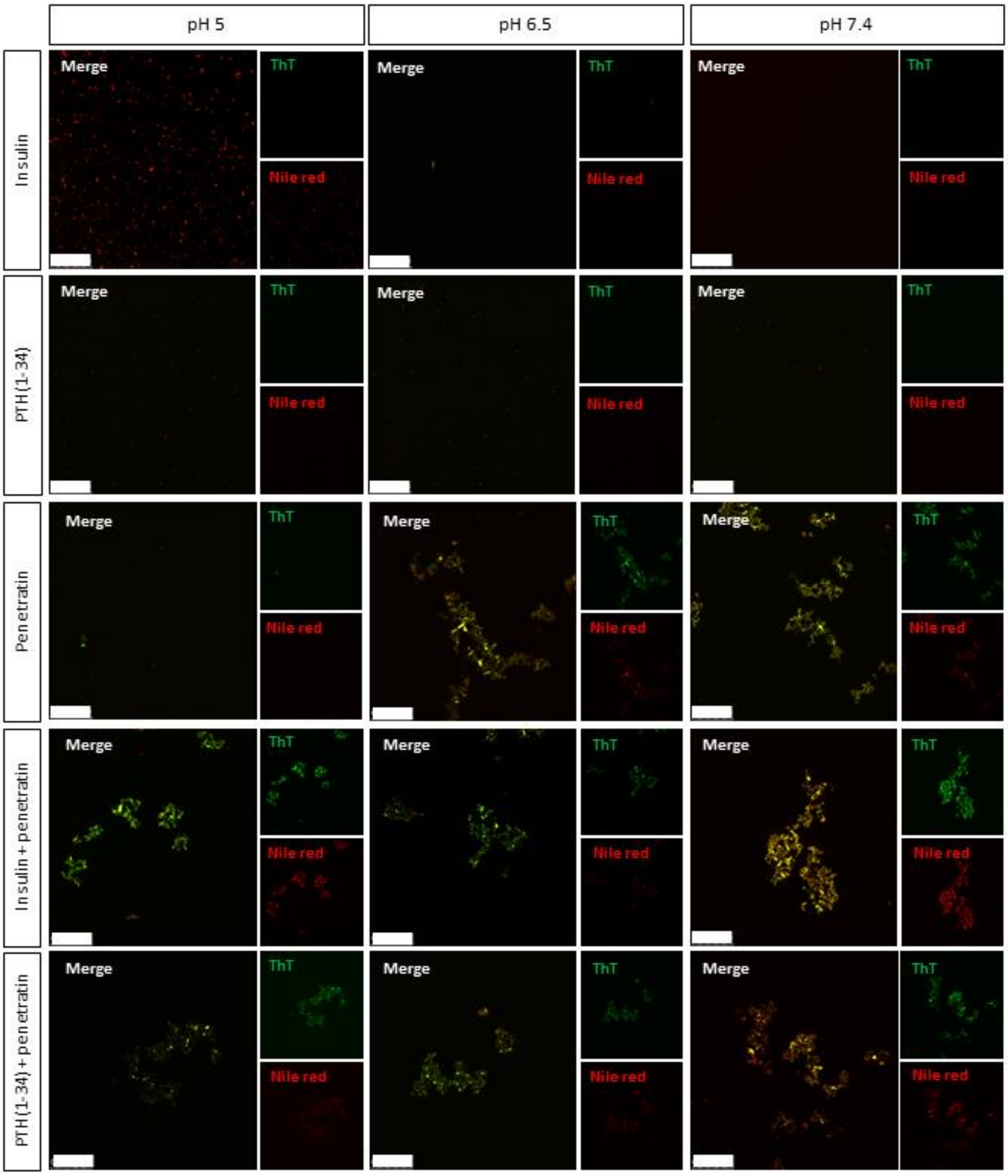

Figure S3. Confocal and two-photon excitation microscopy images of complexes obtained as a result of mixing 50 μM insulin or PTH(1-34) with 200 μM penetratin at pH 5, 6.5, or 7.4 in the presence of Thioflavin T (ThT) (green) and Nile Red (red). Scale bars: 50 μm.

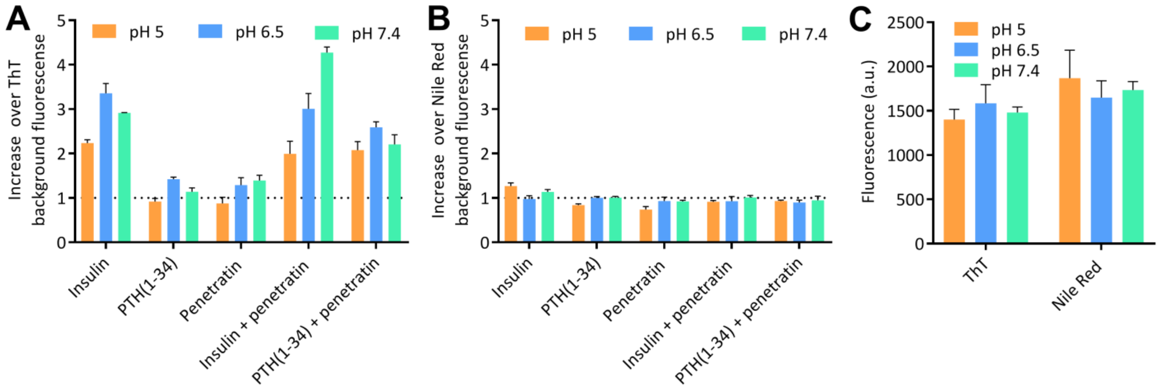

Figure S4. Thioflavin T (ThT) (**A**) and Nile Red (**B**) increase over ThT or Nile Red background fluorescence (**C**) of samples containing 50 µM insulin or PTH(1-34) or 200 µM penetratin alone or as insulin/PTH(1-34) + penetratin mixtures prepared at pH 5, 6.5, or 7.4. (N = 3, mean ± SD).

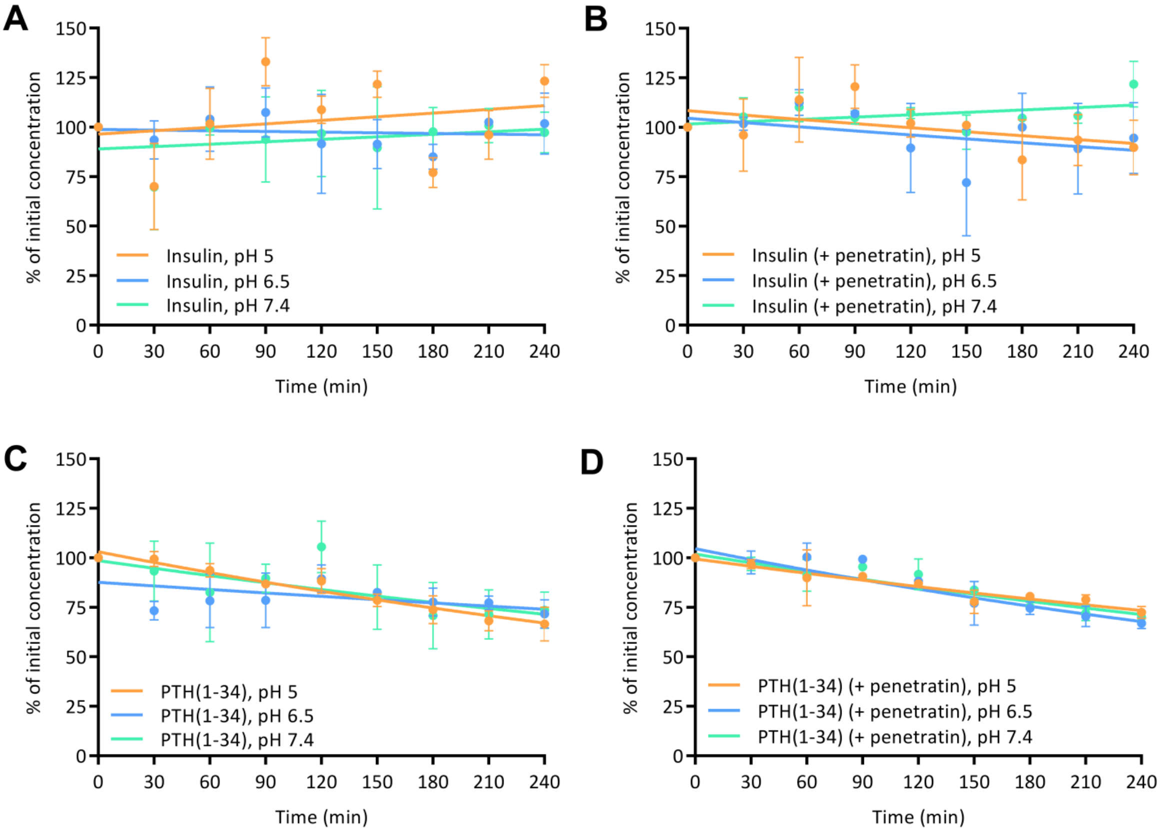

Figure S5. Stability of 5 µM insulin (**A**), 5 µM insulin in the presence of 20 µM penetratin (**B**), 5 µM PTH(1-34) (**C**), or 5 µM PTH(1-34) in the presence of 20 µM penetratin (**D**) during apical incubation on Caco-2 cell monolayers at pH 5, 6.5, or 7.4 over 4 hours. Data are presented as % of initial concentration (N = 3, mean ± SD).

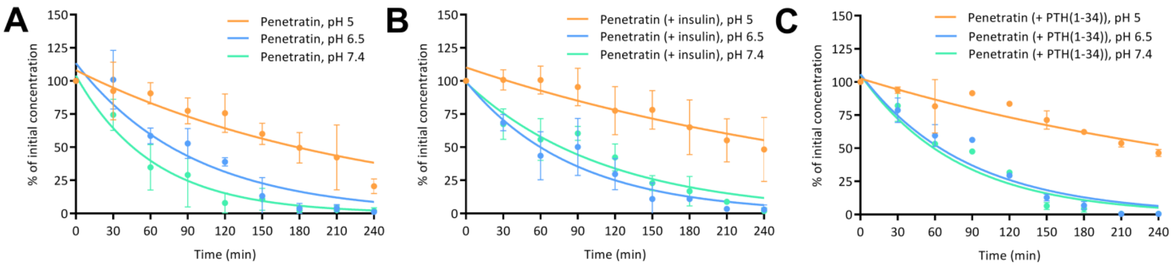

Figure S6. Stability of 20 µM penetratin (**A**), 20 µM penetratin in the presence of 5 µM insulin (**B**), or 20 µM penetratin in the presence of 5 µM PTH(1-34) (**C**) during apical incubation with Caco-2 cell monolayers at pH 5, 6.5, or 7.4 over 4 hours. Data are presented as % of initial concentration ± SD (N = 3, mean ± SD).

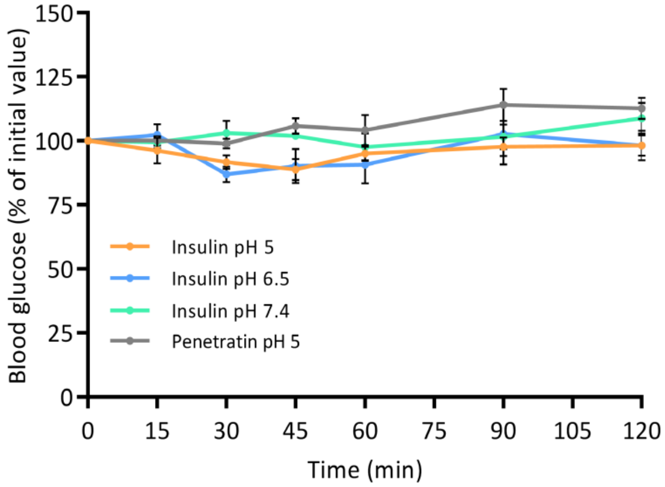

Figure S7. Blood glucose following intraintestinal administration of insulin (50 IU/kg) at pH 5, 6.5, or 7.4 or 720 µM penetratin at pH 5. Data are presented as % of initial value (N = 6, mean ±SD).

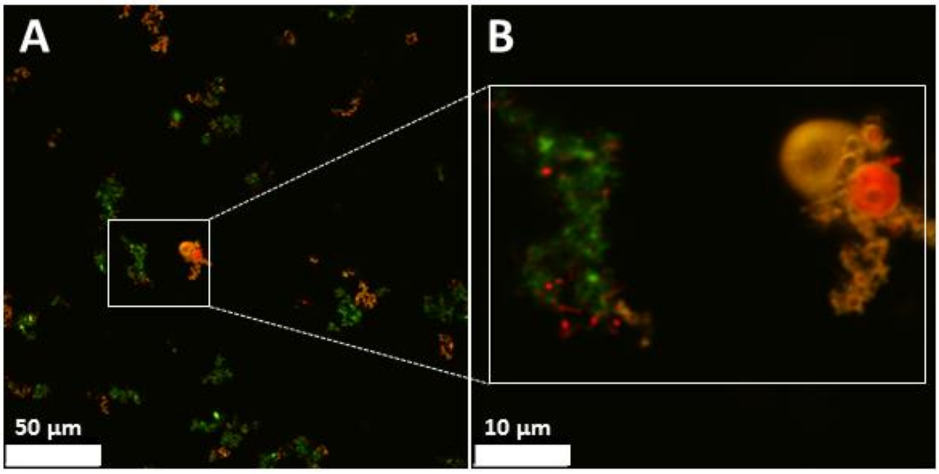

Figure S8. Confocal and two-photon excitation microscopy images of complexes obtained as a result of mixing 50 µM insulin with 200 µM penetratin at pH 6.5 in the presence of POPC:POPG (80:20 molar ratio) liposomes with addition of Thioflavin T (ThT) (green) and Nile Red (red).

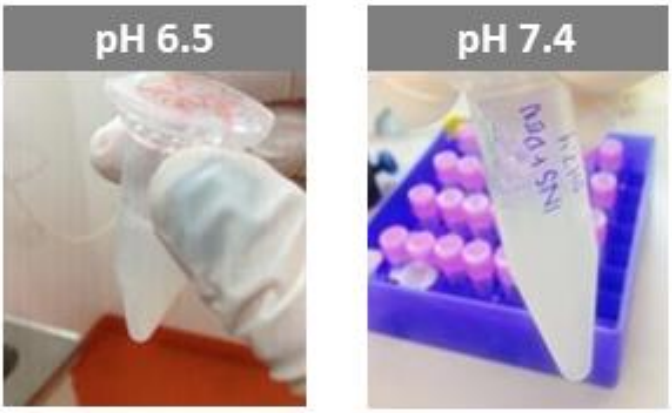

Figure S9. Visual inspection of pH 6.5 (**left**) and pH 7.4 (**right**) samples containing 180 μM insulin in physical mixture with 720 μM penetratin prior intraintestinal administration in rats.

© 2020 by the authors. Licensee MDPI, Basel, Switzerland. This article is an open access article distributed under the terms and conditions of the Creative Commons Attribution (CC BY) license (http://creativecommons.org/licenses/by/4.0/).
